# Weight Status and Body Composition Dynamics in Children and Adolescents During the COVID-19 Pandemic

**DOI:** 10.3389/fped.2021.707773

**Published:** 2021-07-05

**Authors:** Erez Azoulay, Michal Yackobovitch-Gavan, Hadar Yaacov, Inbar Gilboa, Adar Lopez, Tamar Sheppes, Yarden Waksman, Yael Lebenthal, Avivit Brener

**Affiliations:** ^1^Pediatric Endocrinology and Diabetes Unit, Dana-Dwek Children's Hospital, Tel Aviv Sourasky Medical Center, Affiliated With the Sackler Faculty of Medicine, Tel Aviv University, Tel Aviv, Israel; ^2^Department of Epidemiology and Preventive Medicine, School of Public Health, Sackler Faculty of Medicine, Tel Aviv University, Tel Aviv, Israel; ^3^The Nutrition and Dietetics Unit, Dana-Dwek Children's Hospital, Tel Aviv Sourasky Medical Center, Affiliated With the Sackler Faculty of Medicine, Tel Aviv University, Tel Aviv, Israel; ^4^The Psychological Services, Tel Aviv Sourasky Medical Center, Affiliated With the Sackler Faculty of Medicine, Tel Aviv University, Tel Aviv, Israel

**Keywords:** body composition, bioimpedance, BMI, children and adolescents, COVID-19, muscle-to-fat ratio, overweight and obesity, weight status

## Abstract

**Introduction:** The preventive measures taken in attempt to prevent COVID-19 spread lead to closure of schools and leisure time activities. The impact of the pandemic on pediatric weight status is unclear, reports from around the world predict grave consequences with increasing obesity. We aimed to examine the change in body composition parameters of children and adolescents during the pandemic.

**Materials and Methods:** An observational study of 220 pediatric subjects (109 boys; mean current age 11.8 ± 3.3 years; 37 with underweight, 123 with normal weight, and 60 with overweight/obesity) who underwent height and body composition measurements by bioelectrical impedance analysis, Tanita MC-780MA, GMON Professional Software before and during the pandemic. Height, body mass index (BMI) and muscle-to-fat ratio (MFR) z-scores were calculated. Data collected from the participants' medical files included home address for socioeconomic position calculation, pubertal stage, and self-reported sleep duration and physical activity performance.

**Results:** The vast majority of the cohort (81.8%) had stable or improved MFR z-scores during the pandemic. MFR z-scores significantly increased in subjects with underweight (*p* = 0.05) and normal weight (*p* = 0.008), but not in subjects with overweight/obesity (*p* = 0.169). There were significant associations in BMI z-scores (*r* = 0.961, *p* < 0.001) and MFR z-scores (*r* = 0.854, *p* < 0.001) before and during the pandemic. A multivariate linear regression model identified socioeconomic position, pre-pandemic BMI z-scores, pre-pandemic MFR z-scores, and physical activity levels during the pandemic as predictors for delta MFR z-scores (*F* = 12.267, *p* < 0.001). Age, sex, pre-pandemic physical activity, and the time that had elapsed between initiation of the first nationwide lockdown and the BIA assessment during the pandemic did not emerge as predictors for delta MFR z-score.

**Conclusions:** Our encouraging findings demonstrate improvement in body composition parameters of subjects with underweight and normal weight and stability in subjects with overweight/obesity. Engagement in physical activity during the pandemic predicted improvement, while lower socioeconomic position predicted deterioration.

## Introduction

The Coronavirus disease (COVID-19) pandemic continues to demonstrate an enormous impact on morbidity and mortality rates of the entire world population in an age-stratified manner (1–3). On March 15, 2020, the Israeli government declared the first nationwide lockdown, with restriction of non-necessary movement in the public space, and closure of all educational institutions and leisure time activities. The resultant confinement of people to their homes led to lifestyle changes, including diet, sleep, and physical activity. Since then, the Israeli population has been subject to several lockdowns without full restoration of the pre-COVID-19 routine. It has been suggested that this changing reality may have negative implications on metabolic health in adults (4, 5) and in youth (6).

The effect of the COVID-19 pandemic on weight status of children and adolescents remains unclear (7). Reports from around the world predict grave consequences with increasing obesity, especially in predisposed individuals (8–10). Multiple studies are based on lifestyle questionnaires, which subjectively reflect eating behavior and physical activity of adults over a limited time period (11–13), with only a minority of the studies objectively quantitating the effect on the weight status of children (14). Childhood obesity and its future metabolic implications are now of even greater concern than ever with future threats on health economics (15).

Childhood weight status is most widely classified by age- and sex-specific cutoffs for body mass index (BMI) (16). BMI z-scores, however, can not characterize the relationship between fat and muscle (17). Both increased adiposity, especially if centrally located, and decreased skeletal muscle mass have been linked to early-onset metabolic derangements (18–20). Since January 2018, our Pediatric Endocrine Unit implemented the analysis of body composition by means of bioelectrical impedance analysis (BIA) as part of routine assessment of patients referred for endocrine consultation (21). In the current study, we aimed to examine changes in body composition parameters of children and adolescents belonging to different weight status categories (underweight, normal weight, and overweight/obesity), during the COVID-19 pandemic.

## Materials and Methods

### Study Design

This real-life, observational study was conducted in the Pediatric Endocrine Unit in a tertiary medical center. The clinic's BIA database was searched for children and adolescents (age 5–18 years) with the sole diagnosis of “observation of growth” and/or “observation of puberty” whose body composition parameters were measured during the COVID-19 pandemic (from May 15, 2020 until December 15, 2020). Included were subjects for whom pre-COVID-19 BIA measurements were available. Those BIA data were linked to the subjects' electronic medical records. Excluded from the study were patients who entered puberty and those who initiated medication or underwent bariatric surgery between the two time periods.

### Data Collection

Data collected from the participants' medical files included sex, age, home address, perinatal history (mode of conception, number of fetuses, gestational diabetes mellitus, gestational age, and birthweight), anthropometric measurements, systolic (SBP) and diastolic blood pressure (DBP) measurements, pubertal stage, and self-reported sleep duration and physical activity performance.

### Anthropometric Measurements and Pubertal Assessment

The clinical evaluation of the study subjects included measurement of height by a commercial Harpenden stadiometer (Holtain Ltd., Crosswell, United Kingdom) and weight by BIA. BMI was calculated as weight in kilograms divided by height in meters squared. The subjects' height, weight, and BMI values were converted to sex- and age-specific standard deviation scores (z-scores) with PediTools Electronic Growth Chart Calculators, based on CDC growth charts (22). Weight status was categorized as underweight for BMI values ≤ 5th percentile (z-score at or below −1.645), normal for BMI between 5th and 84th percentiles (z-score between −1.645 and 1.036), and overweight and obese for BMI ≥ 85th percentile (z-score at or above 1.036) (23). Corrected birth weight z-scores were calculated with PediTools Electronic Growth Chart Calculators (22). The appropriate for gestational age (AGA) birth weight was defined as corrected birth weight z-scores of −1.88 to 1.88, the small for gestational age (SGA) as birth weight z-scores < −1.88, and the large for gestational age (LGA) as birth weight z-scores > 1.88. BP percentiles were calculated by means of an online age-based pediatric BP calculator (24).

Pubertal stages were graded with Tanner scores for genital status in boys and for breast development in girls (25, 26). Onset of puberty was defined as genitalia Tanner stage 2 with testicular volume > 3 mL in boys and appearance of breast buds in girls, with or without sexual hair. The subject was considered fully pubertal when pubertal signs corresponded to Tanner stage 5.

### Body Composition Measurement

Body composition in subjects older than 5 years of age who were able to stand upright was measured by BIA (Tanita Body Composition Analyzer, Tanita MC-780 MA and GMON Professional Software), which has been clinically verified to be accurate and reliable and to provide highly reproducible results (27, 28). The BIA measures both whole body and segmental analysis (trunk, upper, and lower limbs) of fat and muscle. The BIA measurement required the subject to stand barefoot on the analyzer and to grip the handles, and the entire procedure took ~1 min per subject. The BIA report includes the following data: body weight (kilograms), fat percentage (FATP), truncal fat percentage (TFATP), fat mass (kilograms), and muscle mass (kilograms). Calculated variables included: appendicular skeletal muscle mass (ASMM = the sum of muscle mass of four limbs) and muscle-to-fat ratio [MFR = ASMM (kg)/fat mass (kg)]. The z-scores for MFR were calculated according to BIA pediatric reference curves (29). Delta MFR z-scores were calculated as the difference between MFR z-scores during the COVID-19 pandemic and the MFR z-scores preceding the pandemic. Subjects were stratified into three groups according to delta MFR z-scores: better (delta MFR z-score > 0.3), stable (−0.3 ≤ delta-MFR z-score ≤ 0.3), and worse (delta-MFR z-score < −0.3) (29, 30).

### Socioeconomic Position

The SEP by home address was analyzed based on the Israel Central Bureau of Statistics' Characterization and Classification of Statistical Areas within Municipalities and Local Councils by the Socio-Economic Level of the Population 2015 (31). The neighborhoods or localities are divided into SEP clusters, with 1 representing the lowest and 10 representing the highest rating. The SEP index is an adjusted calculation of 14 variables that measure social and economic levels in the domains of demographics, education, standard of living, and employment (range from −2.797 to 2.590).

### Ethics

The study was approved by the Ethics Committee of the Tel-Aviv Sourasky Medical Center according to the Helsinki Declaration. Informed consent by the participants was waived since the data were retrieved from the subjects' medical records and all personal identification was omitted. The data were handled in accordance with the principles of Good Clinical Practice.

### Statistical Analyses

The data were analyzed with Statistical Package for the Social Sciences software version 27 (SPSS Inc., Chicago, IL). All statistical tests were 2-sided. The Kolmogorov-Smirnov test was applied to test the normality of continuous data. The data are expressed as means ± standard deviations (SDs) for normally distributed variables and median and interquartile range (IQR) for skewed distribution or number (percent) for categorical variables. To compare body composition and BP before and during the COVID-19 pandemic stratified according to weight status, the paired-samples *t*-test or the Wilcoxon matched-pair signed rank-test were used for normal or skewed distributions, respectively. One-way ANOVA analysis and *post-hoc* Tukey or Kruskal Wallis tests were used to compare between change in the MFR ratio z-score categories and weight-status categories (three categories each) in normal and skewed variables, respectively. A one-sample Wilcoxon signed rank test was performed to test the null hypothesis that the median z-score equals zero. Pearson's correlation test was applied to examine the correlation between two continuous data groups. A multivariate stepwise linear regression model was used to identify predictors for the delta MFR z-score. A *p*-value of ≤ 0.05 was considered significant.

## Results

The study cohort was comprised of 220 pediatric subjects (49.5% males) with a mean ± SD current age of 11.8 ± 3.3 years. The median time that had elapsed between BIA assessments (pre-COVID-19 and during COVID-19) was 10.8 months [IQR 7.3, 11.6], and the median time that had elapsed between the initiation of the first nationwide lockdown and the BIA assessment during COVID-19 was 4 months [IQR 3, 5]. The subjects' sociodemographic and perinatal characteristics, pre-COVID-19 anthropometric measurements, BP percentiles, and pubertal status are shown in Table 1. Most of the subjects were conceived spontaneously in a singleton pregnancy and were born at term with birthweight appropriate for gestational age. At the pre-COVID-19 assessment, the median BMI z-score of the cohort was within average (−0.13 [−1.24, 1.14], *p* = 0.383), 45.4% were pre-pubertal, and 54.6% were in puberty/fully pubertal. The median sleep duration was 9 h (range 5–11 h). Most of the subjects (83%) were engaged in some kind of physical activity during and after school.

**Table 1:** Sociodemographic, perinatal, and pre-COVID-19 characteristics of 220 children and adolescents.

**Variable**	
Number of participants	220
Male, *n* (%)	109 (49.5)
Age, years	10.8 ± 3.2
Socioeconomic position cluster	8 [7, 9]
Socioeconomic position index	1.450 [0.825, 1.932]
**Anthropometric characteristics, z-scores**	
Height	−0.62 [−1.37, 0.44]
Weight	−0.47 [−1.63, 1.02]
Body mass index	−0.13 [−1.24, 1.14]
**Blood pressure, percentiles**	
Systolic blood pressure	75 [53, 88]
Diastolic blood pressure	64 [48, 80]
**Pubertal status**, ***n*** **(%)**
Pre-pubertal	100 (45.4)
In puberty	82 (37.3)
Fully pubertal	38 (17.3)
**Perinatal history**	
Assisted reproductive therapy, *n* (%)	12 (5.7)
Gestational diabetes mellitus, *n* (%)	15 (6.8)
Singleton, *n* (%)	206 (93.6)
Preterm birth, *n* (%)	34 (15.5)
Gestational age, weeks	39 [38, 40]
Birth weight, z-scores	−0.41 [−0.91, 0.20]
Small for gestational age, *n* (%)	3 (1.4)
Appropriate for gestational age, *n* (%)	213 (96.8)
Large for gestational age, *n* (%)	4 (1.8)
**Habitual behavior**	
Sleep, hours	9 [8, 10]
**Physical activity**, ***n*** **(%)**	182 (82.7)
None	14 (7.7)
Only at school	17 (9.3)
At and after school	151 (83)

*Data are presented as number (percent), mean ± standard deviation and median [interquartile range]. Socioeconomic position (SEP) was determined by cluster of localities of residence, ranging from 1 to 10, with 1 being the lowest rating and 10 the highest. The SEP index is an adjusted calculation of 14 variables that measure social and economic levels in the domains of demographics, education, standard of living, and employment (ranging from the lowest −2.797 to the highest 2.590). Physical activity was not documented in 38 cases (17.3%) of the study cohort*.

**Figure 1:**
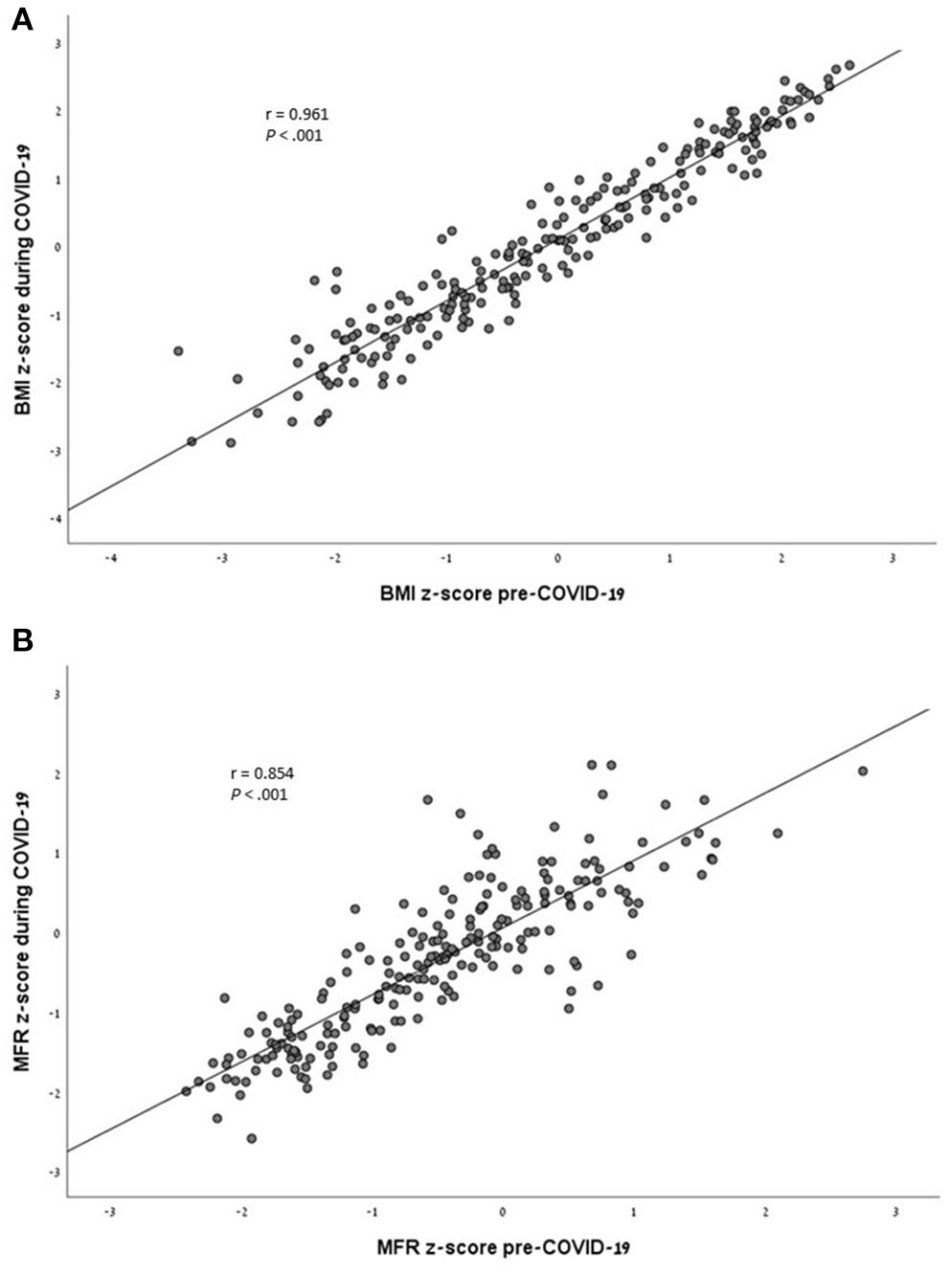
**(A)** Analysis of the correlation between the BMI z-scores of subjects before and during the COVID-19 pandemic (*r* = 0.961, *P* < 0.001). **(B)** Analysis of the correlation between the MFR z-scores of subjects before and during the COVID-19 pandemic (*r* = 0.854, *P* < 0.001).

At the pre-COVID-19 assessment, the cohort was comprised of 37 (16.8%) subjects with underweight, 123 (55.9%) with normal weight, and 60 (27.3%) with overweight/obesity. Subjects with overweight/obesity had a lower median SEP cluster and SEP index compared to those with normal weight and underweight (8 [6.25, 8.75] vs. 8 [8, 9] and 8 [8, 9]; 1.039 [0.422, 1.636] vs. 1.609 [1.002, 1.955] and 1.553 [1.078, 2.005], respectively, *p* < 0.001 for both parameters). The distribution of weight status categories differed between sexes [boys: 26 (23.9%) underweight, 60 (55%) normal weight, and 23 (21.1%) overweight/obesity and girls: 11 (10%) underweight, 93 (56.8%) normal weight, and 37 (33.2%) overweight/obesity, respectively*, p* = 0.009]. The time that had elapsed between the initiation of the first nationwide lockdown and the BIA assessment during the pandemic did not differ between weight categories (*p* = 0.372). During the COVID-19 assessment the cohort was comprised of 26 (11.8%) subjects with underweight, 137 (62.3%) with normal weight, and 57 (25.9%) with overweight/obesity, the change in distribution of weight status categories did not reach significance (*p* = 0.244). The body composition parameters of the cohort stratified by pre-COVID-19 weight status are presented in Table 2. Indices of adiposity, FATP, and TFATP did not significantly change during the pandemic. The MFR z-scores of the underweight and normal weight subjects significantly increased during the pandemic (*p* = 0.05 and *p* = 0.008, respectively), while it did not change in subjects with overweight/obesity (*p* = 0.169). Pearson's correlation analysis revealed significant associations between the BMI z-scores of subjects before and during the pandemic (*r* = 0.961, *p* < 0.001) (Figure 1A), and between the MFR z-scores of subjects before and during the pandemic (0.854, *p* < 0.001) (Figure 1B).

**Table 2:** Body composition and blood pressure characteristics of 220 children and adolescents before and during the COVID-19 pandemic stratified according to weight status.

**Characteristic**	**Before the COVID-19 pandemic**	**During the COVID-19 pandemic**	***p* value**
**Underweight (BMI z-scores** **≤ −1.645)** ***n*** **=** **37 (26 boys)**			
Body mass index, z-scores	−2.07 [−2.34, −1.88]	−1.73 [−2.35, −1.37]	**<0.001**
Fat percentage	17.5 [14.9, 19.9]	17.2 [13.8, 20.1]	0.154
Truncal fat percentage	13.7 [10.8, 15.4]	12.5 [10.0, 15.1]	0.078
Muscle-to-fat, z-scores	−0.08 ± 1.11	0.12 ± 0.95	**0.050**
Systolic BP, percentiles	69 [35, 87]	64 [35, 75]	0.544
Diastolic BP, percentiles	67 [29, 80]	65 [50, 78]	0.168
**Normal weight (−1.645** **<** **BMI z-scores** **<** **1.036)** ***n*** **=** **123 (60 boys)**			
Body mass index, z-scores	−0.38 [−0.95, 0.27]	−0.24 [−0.87, 0.37]	**0.035**
Fat percentage	20.7 [18.2, 24.0]	20.6 [17.1, 24.3]	0.200
Truncal fat percentage	15.5 [13.3, 18.5]	15.3 [11.9, 18.4]	0.091
Muscle-to-fat, z-scores	−0.20 ± 0.84	−0.06 ± 0.81	**0.008**
Systolic BP, percentiles	73 [47, 83]	69 [50, 83]	0.760
Diastolic BP, percentiles	62 [48, 81]	63 [47, 79]	0.783
**Overweight/obesity (BMI z-scores** **≥** **1.036)** ***n*** **=** **60 (23 boys)**			
Body mass index, z-scores	1.74 [1.40, 2.03]	1.70 [1.36, 1.97]	0.412
Fat percentage	33.8 [29.4, 39.2]	33.7 [29.0, 38.5]	0.529
Truncal fat percentage	27.9 [23.7, 34.2]	29.1 [23.6, 33.1]	0.531
Muscle-to-fat, z-scores	−1.42 ± 0.48	−1.36 ± 0.50	0.169
Systolic BP, percentiles	87 [68, 95]	87 [79, 93]	0.122
Diastolic BP, percentiles	72 [53, 82]	75 [58, 86]	0.238

*Data are presented as median [interquartile range] and mean ± SD*.

*Bold indicates statistical significance*.

*BP, blood pressure*.

The vast majority of the cohort (81.8%) had stable or improved MFR z-scores during the pandemic. Sociodemographic, anthropometric, and body composition parameters of the subjects stratified by change in the MFR z-scores (delta MFR z-scores) are presented in Table 3. Sex, age, SEP (cluster and index), and anthropometric parameters did not differ between the delta MFR z-score groups. Subjects whose MFR z-scores worsened during the pandemic had a lower fat percentage, a lower truncal fat percentage, and a higher MFR z-score at baseline (*p* = 0.05, *p* = 0.026, and *p* < 0.001, respectively).

**Table 3:** Sociodemographic, anthropometric, and body composition parameters of the study cohort before COVID-19 pandemic stratified by change in muscle-to-fat ratio (MFR) z-scores.

**Parameter**	**Better**	**Stable**	**Worse**	***p* value**
	**delta-MFR z-score > 0.3**	**−0.3 ≤ delta-MFR z-score ≤ 0.3**	**delta-MFR z-score < −0.3**	
Number	70 (31.8)	110 (50)	40 (18.2)	
Male, *n* (%)	38 (34.9)	51 (46.8)	20 (18.3)	0.583
Age, years	10.3 ± 3.5	10.8 ± 3.1	11.6 ± 2.6	0.114
Socioeconomic position, cluster	8 [7, 9]	8 [7, 9]	8 [7, 9]	0.686
Socioeconomic position, index	1.532 [0.789, 1.985]	1.463 [0.872, 1.919]	1.271 [0.736, 1.748]	0.339
Height, z-scores	−0.67 [−1.39, 0.22]	−0.34 [−1.32, 0.60]	−0.81 [−1.49, 1.04]	0.293
Body mass index, z-scores	−0.44 [−1.49, 0.54]	0.11 [−1.18, 1.46]	−0.04 [−1.24, 1.15]	0.085
Delta body mass index, z-scores	−0.03 [−0.28, 0.18]^a^	0.09 [−0.10, 0.27]^b^	0.29 [−0.02, 0.50]^c^	**<0.001**
Fat percentage	21.5 [18.1, 25.7]^a,b^	23.3 [18.9, 29.8]^b^	20.1 [14.2, 29.2]^a^	**0.050**
Truncal fat percentage	16.8 [13.8, 20.8]^a^	17.6 [14.2, 25.5]^a^	14.7 [9.7, 23.5]^b^	**0.026**
Muscle-to-fat ratio, z-scores	−0.73 ± 0.88^a^	−0.63 ± 0.90^a^	0.16 ± 1.13^b^	**<0.001**
Delta muscle-to-fat ratio, z-scores	0.69 ± 0.37^a^	0.04 ± 0.16^b^	−0.61 ± 0.31^c^	**<0.001**

*The data are expressed as mean ± standard deviation, number (percent) and median [interquartile range]. Socioeconomic position was determined by cluster of localities of residence, ranging from 1 to 10; with 1 being the lowest rating and 10 the highest. The SEP index is an adjusted calculation of 14 variables that measure social and economic levels in the domains of demographics, education, standard of living, and employment (ranging from the lowest −2.797 to the highest 2.590). The values with different superscript letters (a, b) in a column are significantly different from each other in pairwise comparisons (p ≤ 0.05). Bold indicates statistical significance*.

A multivariate linear regression model identified SEP index, pre-pandemic BMI z-score, pre-pandemic MFR z-score, and physical activity during the pandemic as predictors for the delta MFR z-score (*F* = 12.267, *p* < 0.001). Age, sex, pre-pandemic physical activity, and the time that had elapsed between initiation of the first nationwide lockdown and the BIA assessment during the pandemic did not emerge as predictors for delta MFR z-score and therefore were not included in the model (Table 4).

**Table 4:** Linear regression model for delta muscle-to-fat ratio z-scores.

	**Beta**	**Standard error**	***p***
Constant	−0.322	0.104	0.002
SEP index	0.116	0.054	0.033
BMI z-score pre-COVID-19	−0.139	0.030	<0.001
MFR z-score pre-COVID-19	−0.276	0.044	<0.001
Physical activity during COVID-19	0.173	0.086	0.045

*SEP, socioeconomic position; BMI, body mass index; MFR, muscle-to-fat ratio*.

A univariate analysis performed on the findings of the subjects with underweight, normal weight, and overweight/obesity revealed significant correlations between pre-pandemic MFR z-scores and delta MFR z-scores (*r* = −0.524, *p* = 0.001; *r* = −0.391, *p* < 0.001 and *r* = −0.291, *p* = 0.024, respectively, and *p* = 0.428 for the comparison between correlations).

## Discussion

It could be reasonably expected that the extended period out of school would lead to an increase in sedentary behavior with worsening of body composition. However, the subjects in our select cohort tended to maintain similar BMI and MFR z-scores, and 31.8% even improved their body composition during the COVID-19 pandemic. Improvement in both weight status (BMI z-scores) and body composition (MFR z-scores) was evidenced in subjects with underweight and normal weight, while those with overweight/obesity maintained stable parameters. Engagement in physical activity during the pandemic predicted an improvement in body composition, while lower socioeconomic position predicted deterioration.

The COVID-19 pandemic has had enormous effects on lifestyle that could potentially cause health-related problems. In Israel, most workplaces gradually returned to full activity under strict safety guidance. Schools were the last to resume activity, with limited numbers of schools reopening (kindergartens, grades 1–3 and 11–12) since May 2020. Major COVID-19 outbreaks were reported in schools only a few days after opening, leading to a second unlimited closure of all educational settings (32). The exclusion of the pediatric population from the vaccination program was an obstacle in planning the reopening of school (33). Two studies suggested that the prolonged closure of schools may disproportionately affect activity patterns and weight status of youths (34, 35). Of note, 92.3% of our cohort had been engaged in physical activity during school hours in the pre-pandemic period, and 83% also did so after school hours. Encouragement in participating in physical activity is an integral part of the healthy lifestyle education provided to all the subjects followed at our clinic, regardless of their weight status. Our results could, therefore, be attributed in no small part to the increased awareness of healthy habits and the desirability of maintaining them. Another potential contributor is the increased accessibility and popularity of at-home physical activity through web-based applications (YouTube, Tik-Tok, etc.). Surveys for quantification of physical activity were not part of this observational study.

The extent of change in body composition differed between weight status categories, with the greatest improvement measured in the subjects with underweight. The worse their pre-pandemic body composition, the greater the improvement that had been measured during the pandemic. Children and adolescents with low weight status are considered at increased risk for infectious diseases (36, 37). The pandemic rampage with increasing anxiety of developing severe manifestation might have raised the parental motivation for improving the offspring's nutrition status. In addition, the prolonged stay at home changed familial practices with increased time spent in planning and preparing meals (38). Of note, subjects with underweight did not only improve their BMI z-scores as a result of increased caloric consumption; their body composition improvement may reflect a healthier food selection combined with physical activity. This improvement in muscle mass may decrease the metabolic hazards related to early-onset sarcopenia (39). The combination of sarcopenia with increased adiposity, termed “sarcopenic obesity,” may result in the false impression of slightly elevated BMI while harboring a double jeopardy (40).

The management of pediatric obesity is a daunting challenge, especially since lifestyle interventions have shown only modest effect on weight loss (41). The COVID-19 period further intensified the challenge faced by obese children who were threatened by increased obesity and metabolic health deterioration (42). We were gratified to observe that, on average, weight status, body composition, and BP percentiles of subjects with overweight and obesity were stable. This could not be attributed to any new medication or their having undergone bariatric surgery, since they were study exclusion criteria. A plausible explanation for this welcome achievement could be the preservation of continuity of surveillance in the clinic in spite of the ongoing pandemic. Our multidisciplinary team of physicians, nurse educators, dieticians, and psychosocial workers deliver age-appropriate medical care and healthy lifestyle education to clinic attendees and their families. The continuity of medical care, either in-clinic or by telemedicine, provides a network of support that is crucial in obesity management (43).

A lower socioeconomic position predicted decreased delta MFR z-score during the COVID-19 pandemic. Socioeconomic circumstances are recognized as a major determinant of health conditions in children and adolescents (44). The COVID-19 pandemic has led to an economic crisis, characterized by increasing rates of unemployment and financial insecurity with increasing health inequalities (45). Coping with financial strain may negatively impact health behaviors, such as leading to unhealthy food choices and decreased enthusiasm for physical activity (46). While our society as a whole was not immune to such financial upheavals, our patients were apparently successfully persuaded not to fall victim to such consequences. The metabolic consequences of these will be dealt in the future, as unhealthy lifestyle during childhood has both metabolic and behavioral effects (47). Health care systems should take these findings into consideration in planning contemporary health care strategies.

Our study is not without limitations. The cohort was comprised of children and adolescents, both boys and girls, from a wide age range, at various stages of physical growth and development. Cognitive and emotional maturation depends on age and the course of development may vary between the sexes. Therefore, our ability to draw conclusion on health behaviors of a specific age group during the pandemic is limited. In addition, our study did not include questionnaires for evaluating nutritional, behavioral and psychological aspects before and during the COVID-19 pandemic. Since only patients who attended our pediatric endocrine clinic during the pandemic were included, the results may not reflect the body composition of individuals who are not similarly followed by professional medical care providers. Therefore, the generalizability of this study may be limited. Nevertheless, our encouraging findings support the recommendations for global growth surveillance, including a healthy lifestyle education (48). This undertaking is crucial in the current challenging period of spiraling obesity. The major strength of this study is the uniformity of anthropometric, body composition, and BP measurements, together with the calculation of sex- and age-adjusted z-scores/percentiles, allowing for comparisons between subjects.

## Conclusion

The weight status and body composition of children and adolescents attending our pediatric endocrine clinic were relatively stable during the COVID-19 pandemic. Subjects with underweight and normal weight had improved body composition parameters, while those with overweight/obesity remained stable. Engagement in physical activity during the pandemic predicted an improvement in body composition, while lower socioeconomic position predicted deterioration. These encouraging findings may well be attributed to the regular growth surveillance and healthy lifestyle education provided to the study participants. We suggest that their availability to the pediatric population at large will produce a similarly rewarding impact on health and far-reaching positive repercussions on health economics.

## Data Availability Statement

The raw data supporting the conclusions of this article will be made available by the authors, without undue reservation.

## Ethics Statement

The studies involving human participants were reviewed and approved by Tel Aviv Sourasky Medical Center. Written informed consent from the participants' legal guardian/next of kin was not required to participate in this study in accordance with the national legislation and the institutional requirements.

## Author Contributions

EA contributed, analyzed and interpreted the data, wrote the first draft, and revised the manuscript. MY-G performed the statistical analysis, interpreted the data, and critically revised the manuscript. HY, IG, AL, TS, and YW contributed to the data used in this study, contributed to the discussion, and reviewed and edited the manuscript. YL conceived and designed the study, interpreted the data, and revised the manuscript for important intellectual content. AB conceived and designed the study, analyzed and interpreted the data, wrote the first draft and revised the manuscript, incorporating contributions from coauthors (EA, MY-G, HY, IG, AL, TS, YW, and YL), and decided on submission. AB is the guarantor of this work and as such, had full access to all the data in the study and takes responsibility for the integrity of the data and the accuracy of the data analysis. All authors contributed to the article and approved the submitted version.

## Conflict of Interest

The authors declare that the research was conducted in the absence of any commercial or financial relationships that could be construed as a potential conflict of interest.

## Abbreviations

ASMM: appendicular skeletal muscle mass
BIA: bioimpedance analysis
BMI: body mass index
FATP: fat percentage
TFATP: truncal fat percentage
MFR: muscle-to-fat ratio
MPHt: mid-parental height
SEP: socioeconomic position.

## References

[1] Banerjee A Pasea L Harris S Gonzalez-Izquierdo A Torralbo A Shallcross L. Estimating excess 1-year mortality associated with the COVID-19 pandemic according to underlying conditions and age: a population-based cohort study. Lancet. (2020) 395:1715–25. 10.1016/S0140-6736(20)30854-0 32405103 PMC7217641

[2] Li Q Guan X Wu P Wang X Zhou L Tong Y. Early transmission dynamics in Wuhan, China, of novel coronavirus-infected pneumonia. N Engl J Med. (2020) 382:1199–207. 10.1056/NEJMoa2001316 31995857 PMC7121484

[3] Puig-Domingo M Marazuela M Giustina A. COVID-19 and endocrine diseases. A statement from the European Society of Endocrinology. Endocrine. (2020) 68:2–5. 10.1007/s12020-020-02294-5 32279224 PMC7150529

[4] Martinez-Ferran M de la Guía-Galipienso F Sanchis-Gomar F Pareja-Galeano H. Metabolic impacts of confinement during the COVID-19 pandemic due to modified diet and physical activity habits. Nutrients. (2020) 12:1549. 10.3390/nu12061549 32466598 PMC7352228

[5] Wang G Zhang Y Zhao J Zhang J Jiang F. Mitigate the effects of home confinement on children during the COVID-19 outbreak. Lancet. (2020) 395:945–7. 10.1016/S0140-6736(20)30547-X 32145186 PMC7124694

[6] An R. Projecting the impact of the coronavirus disease-2019 pandemic on childhood obesity in the United States: a microsimulation model. J Sport Health Sci. (2020) 9:302–12. 10.1016/j.jshs.2020.05.006 32454174 PMC7250129

[7] Cuschieri S Grech S. COVID-19: a one-way ticket to a global childhood obesity crisis? J Diabetes Metab Disord. (2020) 19:1–4. 10.1007/s40200-020-00682-2 33173756 PMC7644278

[8] Pietrobelli A Pecoraro L Ferruzzi A Heo M Faith M Zoller T. Effects of COVID-19 lockdown on lifestyle behaviors in children with obesity living in Verona, Italy: a longitudinal study. Obesity. (2020) 28:1382–5. 10.1002/oby.22861 32352652 PMC7267384

[9] Robinson E Boyland E Chisholm A Harrold J Maloney NG Marty L. Obesity, eating behavior and physical activity during COVID-19 lockdown: a study of UK adults. Appetite. (2021) 156:104853. 10.1016/j.appet.2020.104853 33038479 PMC7540284

[10] Rundle AG Park Y Herbstman JB Kinsey EW Wang YC. COVID-19-related school closings and risk of weight gain among children. Obesity. (2020) 28:1008–9. 10.1002/oby.22813 32227671 PMC7440663

[11] Reyes-Olavarría D Latorre-Román PÁ Guzmán-Guzmán IP Jerez-Mayorga D Caamaño-Navarrete F Delgado-Floody P. Positive and negative changes in food habits, physical activity patterns, and weight status during COVID-19 confinement: associated factors in the Chilean population. Int J Environ Res Public Health. (2020) 17:5431. 10.3390/ijerph17155431 32731509 PMC7432624

[12] Sidor A Rzymski P. Dietary choices and habits during COVID-19 lockdown: experience from Poland. Nutrients. (2020) 12:1–13. 10.3390/nu12061657 32503173 PMC7352682

[13] Kriaucioniene V Bagdonaviciene L Rodríguez-Pérez C Petkeviciene J. Associations between changes in health behaviours and body weight during the COVID-19 quarantine in Lithuania: the Lithuanian COVIDiet study. Nutrients. (2020) 12:3119. 10.3390/nu12103119 33065991 PMC7599784

[14] Tester JM Rosas LG Leung CW. Food insecurity and pediatric obesity: a double whammy in the era of COVID-19. Curr Obes Rep. (2020) 9:442–50. 10.1007/s13679-020-00413-x 33064269 PMC7562757

[15] Ogden CL Fryar CD Hales CM Carroll MD Aoki Y Freedman DS. Differences in obesity prevalence by demographics and urbanization in US children and adolescents, 2013-2016. JAMA. (2018) 319:2410–8. 10.1001/jama.2018.5158 29922826 PMC6393914

[16] Cole TJ Bellizzi MC Flegal KM Dietz WH. Establishing a standard definition for child overweight and obesity worldwide: international survey. BMJ. (2000) 320:1240–3. 10.1136/bmj.320.7244.1240 10797032 PMC27365

[17] Maynard LM Wisemandle W Roche AF Chumlea WC Guo SS Siervogel RM. Childhood body composition in relation to body mass index. Pediatrics. (2001) 107:344–50. 10.1542/peds.107.2.344 11158468

[18] Kyle UG Earthman CP Pichard C Coss-Bu JA. Body composition during growth in children: limitations and perspectives of bioelectrical impedance analysis. Eur J Clin Nutr. (2015) 69:1298–305. 10.1038/ejcn.2015.86 26039314

[19] Maffeis C Morandi A. Body composition and insulin resistance in children. Eur J Clin Nutr. (2018) 72:1239–45. 10.1038/s41430-018-0239-2 30185840

[20] Steene-Johannessen J Anderssen SA Kolle E Andersen LB. Low muscle fitness is associated with metabolic risk in youth. Med Sci Sports Exerc. (2009) 41:1361–7. 10.1249/MSS.0b013e31819aaae5 19516166

[21] Brener A Peleg I Rosenfeld T Kern S Uretzky A Elkon-Tamir. Beyond body mass index - body composition assessment by bioimpedance in routine endocrine practice. Endocr Pract. (2021) 27:419–25. 10.1016/j.eprac.2020.10.013 33934752

[22] Chou JH Roumiantsev S Singh R. PediTools electronic growth chart calculators: applications in clinical care, research, and quality improvement. J Med Internet Res. (2020) 22:e16204. 10.2196/16204 32012066 PMC7058170

[23] Barlow SE Expert Committee. Expert committee recommendations regarding the prevention, assessment, and treatment of child and adolescent overweight and obesity: summary report. Pediatrics. (2007) 120 (Suppl. 4):S164–92. 10.1542/peds.2007-2329C 18055651

[24] Shypailo RJ. Age-Based Pediatric Blood Pressure Reference Charts. Retrieved 1/5/2021 from the Baylor College of Medicine, Children's Nutrition Research Center, Body Composition Laboratory. (2018). Available online at: http://www.bcm.edu/bodycomplab/BPappZjs/BPvAgeAPPz.html (accessed April 6, 2021).

[25] Marshall WA Tanner JM. Variations in pattern of pubertal changes in girls. Arch Dis Child. (1969) 44:291–303. 10.1136/adc.44.235.291 5785179 PMC2020314

[26] Marshall WA Tanner JM. Variations in the pattern of pubertal changes in boys. Arch Dis Child. (1970) 45:13–23. 10.1136/adc.45.239.13 5440182 PMC2020414

[27] Lee SY Gallagher D. Assessment methods in human body composition. Curr Opin Clin Nutr Metab Care. (2008) 11:566–72. 10.1097/MCO.0b013e32830b5f23 18685451 PMC2741386

[28] Shypailo RJ Motil KJ. The use of bioimpedance in pediatric health, nutrition, and disease. J Pediatr Gastroenterol Nutr. (2018) 67:435–6. 10.1097/MPG.0000000000002068 30239486

[29] McCarthy HD Samani-Radia D Jebb SA Prentice AM. Skeletal muscle mass reference curves for children and adolescents. Pediatr Obes. (2014) 9:249–59. 10.1111/j.2047-6310.2013.00168.x 23776133

[30] Mei Z Grummer-Strawn LM. Standard deviation of anthropometric Z-scores as a data quality assessment tool using the 2006 WHO growth standards: a cross country analysis. Bull World Health Organ. (2007) 85:441–8. 10.2471/BLT.06.034421 17639241 PMC2636355

[31] Israel Central Bureau of Statistics (CBS); 2019. Characterization and Classification of Geographical Units by the Socio-Economic Level of the Population. (2015). Available online at: https://www.cbs.gov.il/he/publications/DocLib/2019/1765_socio_economic_2015/e_print.pdf (accessed March 1, 2021).

[32] Stein-Zamir C Abramson N Shoob H Libal E Bitan M Cardash T. A large COVID-19 outbreak in a high school 10 days after schools' reopening, Israel, May 2020. Euro Surveill. (2020) 25:2001352. 10.2807/1560-7917.ES.2020.25.29.2001352 32720636 PMC7384285

[33] Dagan N Barda N Kepten E Miron O Perchik S Katz MA. BNT162b2 mRNA Covid-19 vaccine in a nationwide mass vaccination setting. N Engl J Med. (2021) 384:1412–23. 10.1056/NEJMoa2101765 33626250 PMC7944975

[34] Yang S Guo B Ao L Yang C Zhang L Zhou J. Obesity and activity patterns before and during COVID-19 lockdown among youths in China. Clin Obes. (2020) 10:e12416. 10.1111/cob.12416 33009706 PMC7646045

[35] Jia P Zhang L Yu W Yu B Liu M Zhang D. Correction: impact of COVID-19 lockdown on activity patterns and weight status among youths in China: the COVID-19 impact on lifestyle change survey (COINLICS). Int J Obes. (2021) 45:920. 10.1038/s41366-020-00736-8 33580192 PMC7880021

[36] Walson JL Berkley JA. The impact of malnutrition on childhood infections. Curr Opin Infect Dis. (2018) 31:231–236. 10.1097/QCO.0000000000000448 29570495 PMC6037284

[37] Mertens E Peñalvo JL. The burden of malnutrition and fatal COVID-19: a global burden of disease analysis. Front Nutr. (2021) 7:619850. 10.3389/fnut.2020.619850 33553234 PMC7858665

[38] Philippe K Chabanet C Issanchou S Monnery-Patris S. Child eating behaviors, parental feeding practices and food shopping motivations during the COVID-19 lockdown in France: (How) did they change? Appetite. (2021) 161:105132. 10.1016/j.appet.2021.105132 33493611 PMC7825985

[39] Ooi PH Thompson-Hodgetts S Pritchard-Wiart L Gilmour SM Mager DR. Pediatric sarcopenia: a paradigm in the overall definition of malnutrition in children? J Parenter Enteral Nutr. (2020) 44:407–18. 10.1002/jpen.1681 31328301

[40] Cauley JA. An overview of sarcopenic obesity. J Clin Densitom. (2015) 18:499–505. 10.1016/j.jocd.2015.04.013 26141163

[41] Kumar S Kelly AS. Review of childhood obesity: from epidemiology, etiology, and comorbidities to clinical assessment and treatment. Mayo Clin Proc. (2017) 92:251–65. 10.1016/j.mayocp.2016.09.017 28065514

[42] Nogueira-de-Almeida CA Del Ciampo LA Ferraz IS Del Ciampo IRL Contini AA Ued FDV. COVID-19 and obesity in childhood and adolescence: a clinical review. J Pediatr. (2020) 96:546–58. 10.1016/j.jped.2020.07.001 32768388 PMC7402231

[43] O'Hara VM Johnston SV Browne NT. The paediatric weight management office visit via telemedicine: pre- to post-COVID-19 pandemic. Pediatr Obes. (2020) 15:e12694. 10.1111/ijpo.12694 32627434 PMC7361154

[44] Moreno-Maldonado C Ramos P Moreno C Rivera F. Direct and indirect influences of objective socioeconomic position on adolescent health: the mediating roles of subjective socioeconomic status and lifestyles. Int J Environ Res Public Health. (2019) 16:1637. 10.3390/ijerph16091637 31083434 PMC6539554

[45] Wright L Steptoe A Fancourt D. Are we all in this together? Longitudinal assessment of cumulative adversities by socioeconomic position in the first 3 weeks of lockdown in the UK. J Epidemiol Community Health. (2020) 74:683–8. 10.1136/jech-2020-214475 32503892 PMC7298206

[46] Beenackers MA Oude Groeniger J van Lenthe FJ Kamphuis CBM. The role of financial strain and self-control in explaining health behaviours: the GLOBE study. Eur J Public Health. (2018) 28:597–603. 10.1093/eurpub/ckx212 29236973 PMC6051441

[47] Wang J Geng L. Effects of socioeconomic status on physical and psychological health: lifestyle as a mediator. Int J Environ Res Public Health. (2019) 16:281. 10.3390/ijerph16020281 30669511 PMC6352250

[48] Lobstein T Jackson-Leach R Moodie ML Hall KD Gortmaker SL Swinburn BA. Child and adolescent obesity: part of a bigger picture. Lancet. (2015) 385:2510–20. 10.1016/S0140-6736(14)61746-3 25703114 PMC4594797

